# Effectiveness of *TaDreb-B1* and *1-FEH w3* KASP Markers in Spring and Winter Wheat Populations for Marker-Assisted Selection to Improve Drought Tolerance

**DOI:** 10.3390/ijms24108986

**Published:** 2023-05-19

**Authors:** Shamseldeen Eltaher, Mostafa Hashem, Asmaa A. M. Ahmed, P. Stephen Baenziger, Andreas Börner, Ahmed Sallam

**Affiliations:** 1 Department of Plant Biotechnology, Genetic Engineering and Biotechnology Research Institute (GEBRI), University of Sadat City (USC), Sadat City 32897, Egypt; shams.eltaher@gebri.usc.edu.eg; 2 Department of Genetics, Faculty of Agriculture, Assiut University, Assiut 71526, Egypt; mostafa.14224423@agr.aun.edu.eg (M.H.); asmaa.14219580@edu.aun.edu.eg (A.A.M.A.); 3 Department of Agronomy & Horticulture, University of Nebraska-Lincoln, Lincoln, NE 68583, USA; pbaenziger1@unl.edu; 4 Department Genebank, Leibniz Institute of Plant Genetics and Crop Plant Research (IPK), 06466 Gatersleben, Germany

**Keywords:** kompetitive allele-specific PCR, genetic validation, water deficit, MAS

## Abstract

Due to the advances in DNA markers, kompetitive allele-specific PCR (KASP) markers could accelerate breeding programs and genetically improve drought tolerance. Two previously reported KASP markers, *TaDreb-B1* and *1-FEH w3*, were investigated in this study for the marker-assisted selection (MAS) of drought tolerance. Two highly diverse spring and winter wheat populations were genotyped using these two KASP markers. The same populations were evaluated for drought tolerance at seedling (drought stress) and reproductive (normal and drought stress) growth stages. The single-marker analysis revealed a high significant association between the target allele of *1-FEH w3* and drought susceptibility in the spring population, while the marker–trait association was not significant in the winter population. The *TaDreb-B1* marker did not have any highly significant association with seedling traits, except the sum of leaf wilting in the spring population. For field experiments, SMA revealed very few negative and significant associations between the target allele of the two markers and yield traits under both conditions. The results of this study revealed that the use of *TaDreb-B1* provided better consistency in improving drought tolerance than *1-FEH w3*.

## 1. Introduction

Wheat (*Triticum aestivum* L.) is the main source of nutrition for one-third of the world’s population [1]. It is now necessary, despite limited resources, to improve wheat production to fulfill the demand for food. To meet this need, the breeding of high-yielding cultivars is essential. Years of conventional and molecular breeding in wheat have greatly improved wheat yield. Understanding genetic principles, phenotypic evaluation, and selection through traditional breeding methods has resulted in significant improvements in wheat productivity [2,3]. However, increasing productivity in the face of global warming and weather variability will be a difficult task. Due to unpredictable crop loss, abiotic stresses influence wheat yield and can lead to a food supply deficit. Drought and heat are the two most significant abiotic factors that limit wheat yield [4,5,6]. Many researchers have identified drought tolerance in wheat over the past few decades, but they have not been able to improve the crops’ drought tolerance due to a variety of causes. First, the diverse changes in several physiological parameters of the plant caused by drought need to be quantified and better understood. Second, the selection process is impacted by the genotype x environment interaction (GE), where the environment is often variable. Third, drought is a very complicated feature that is regulated by numerous genes, the majority of which contribute only minimally genetically. To improve wheat’s ability to tolerate drought, all of these three reasons should be taken into consideration. Additionally, the complexity of the wheat genome is one of the most significant elements reducing the effectiveness of the process of increasing drought tolerance in wheat.

The wheat breeding program aims to screen as many as wheat genotypes for their tolerance to drought to select promising drought-tolerant genotypes that can be crossed to produce wheat cultivars that have a high tolerance to drought stress. However, phenotypic evaluation is labor and takes a lot of time to achieve [7,8]. DNA markers linked to target traits can be used through marker-assisted selection (MAS) to accelerate breeding programs. The development of molecular markers has three main limitations: (1) a functional marker must be developed based on the gene sequence, or the markers must be tightly linked or co-segregated with the genes controlling the traits; (2) they should also be broadly applicable to various cultivars and geographical areas; and (3) when used in breeding programs, the markers should be inexpensive [9]. Unfortunately, most traditional molecular markers, including those developed over the past 30 years (such as RAPD, AFLP, SSR, and RFLP) are not on target gene sequences and frequently exist at high genetic distances from genes. In addition, cultivars showing desired marker genotypes may not necessarily have the targeted genes—a phenomenon known as “false positives” in MAS practice. Additionally, while choosing markers, many markers have low polymorphism [9,10].

Single nucleotide polymorphisms (SNPs) are base pair substitutions, deletions, or insertions that occur as point mutations in a genome. An efficient, homogenous, and fluorescence-based method called kompetitive allele-specific PCR (KASP) genotyping allows for the detection and calculation of SNPs such as insertions and deletions [11,12,13]. KASP is a uniplex and flexible genotyping platform that efficiently and cheaply provides high throughput [14]. Breeding program improvement could be greatly sped up by converting traditional functional markers (FMs) to KASP tests.

KASP is rarely used to investigate the effects of drought on wheat at the seedling stage, two KASP markers, namely *TaDreb-B1* and *1-FEH w3* from *Dreb* and *Fehw3* genes, were reported with their association with drought tolerance [15]. Only two studies tested these markers with their association with yield under normal conditions and drought tolerance at the germination stage, and those studies were by Rehman et al. [16] and Mohamed et al. [17], respectively. Therefore, insufficient information is provided about these two markers for drought tolerance to be used in MAS, in which big germplasm can be screened. Validating DNA markers is an important step before utilizing them in MAS to achieve fruitful improvement for target traits. Such validation of markers should be significantly tested in a different genetic background, or their effects can still be significantly detected when tested in different locations and/or years [18]. Moreover, the marker–trait association should be tested in diverse populations that have high genetic variation, which is very important to truly investigate the association of the tested markers with the target traits for MAS. The aims of this study are to test the association between drought tolerance and two KASP markers (*TaDreb-B1* and *1-FEH w3* markers) and investigate their effectiveness for marker-assisted selection to improve drought tolerance in wheat.

## 2. Results

### 2.1. KASP Genotyping

**Figure 1:**
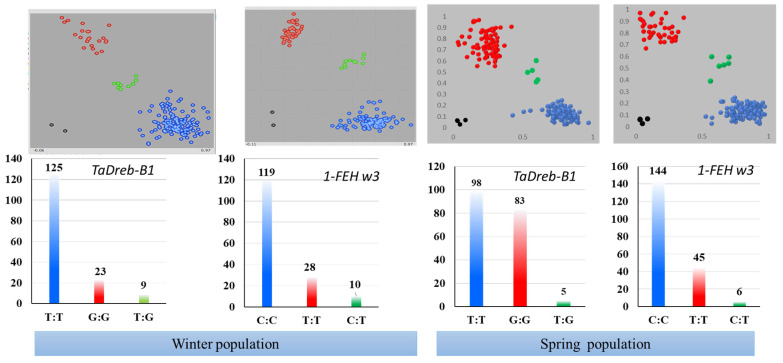
The result of KASP genotyping in spring and winter populations using *TaDreb-B1* and *1-FEH w3* KASP markers. T:T (blue) and G:G (red) homozygous allele and T:G (green) heterozygous allele for *TaDreb-B1*. C:C (blue) and T:T (red) homozygous allele and C:T (green) heterozygous allele for *1-FEH w3*.

The distribution of two wheat populations based on *TaDreb-B1* and *1-FEH w3* KASP markers is shown in Figure 1. In general, the blue dots are the FAM- homozygous allele and refer to the marker’s target allele. The red dots are the HEX homozygous allele (non-target allele). Green dots are heterozygous alleles, and black dots stand for no call.

According to Figure 1, the winter population (WP) had 125 genotypes containing the homozygous allele (T:T) for *TaDreb-B1* and 119 genotypes containing the homozygous allele (C:C) for *1-FEH w3*. Additionally, the red groups in the WP containing 23 genotypes had homozygous alleles (G:G) for *TaDreb-B1*, and 28 genotypes had homozygous alleles (T:T) for *1-FEH w3*. The heterozygous alleles (T:G and C:T) in the WP were shown in 9 and 10 genotypes for *TaDreb-B1* and *1-FEH w3*, respectively.

The spring population (SP) had 98 genotypes with the homozygous allele (T:T) in the *TaDreb-B1* and 144 genotypes with the homozygous allele (C:C) in the *1-FEH w3*. Additionally, 83 genotypes had the homozygous allele (G:G) for *TaDreb-B1*, and 45 genotypes had the homozygous allele (T:T) for *1-FEH w3* in the red groups. The heterozygous alleles (T:G and C:T) in the SP were present in 5 and 6 genotypes for *TaDreb-B1* and *1-1-FEH w3*, respectively.

**Figure 2:**
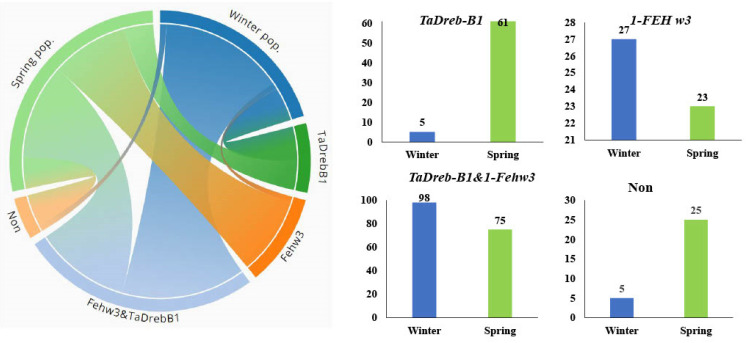
Number of genotypes having only gene; only *TaDreb-B1* allele, only *1-FEH w3* allele, both genes (TaDreb-B1 and 1-FEH w3) and no target allele (Non group).

The genotypes in each population were classified into four groups: *TaDrebB1* (genotypes had only the *TaDreb-B1* target allele), *1-FEH w3* (genotype had only the *1-FEH w3* target allele), *TaDrebB1*&*1-FEH w3* (genotype had the two target alleles), and Non (genotype did not have any target alleles) (Figure 2). The spring population had 61 genotypes with only TaDreB1, and this was higher than those genotypes in the winter population (5 genotypes). For genotypes having only *1-FEH w3*, a set of 27 genotypes in the winter population was found to have only *1-FEH w3*, and this number was higher than those in the spring population (23 genotypes). Interestingly, 62% (98 genotypes) of genotypes in the winter population possessed both favorable marker alleles together, while this percentage was only 37% in the spring population (75 genotypes). A total of 25 genotypes had no favorable marker allele in the spring genotypes, while only five genotypes in the winter population had no favorable marker allele.

### 2.2. The Diversity of TaDreb-B1 and 1-FEH w3 Markers

**Figure 3:**
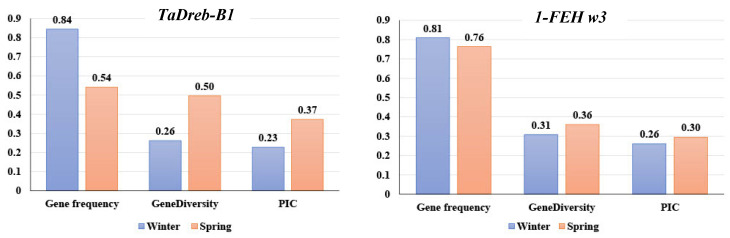
Diversity features. Gene frequency, gene diversity, and polymorphism information content (PIC) of *TaDreb-B1* and *1-FEH w3* markers in each population.

The marker allele frequency, marker allele diversity, and PIC for both marker alleles in the two wheat populations are presented in Figure 3. In the WP and SP, the *TaDreb-B1* gene frequency was detected at 0.84 and 0.54, respectively. Moreover, the gene diversity of *TaDreb-B1* in the WP and SP was 0.26 and 0.50, respectively. PIC values for *TaDreb-B1* were 0.23 and 0.37 in WP and SP, respectively.

The gene frequency of *1-FEH w3* was 0.81 in the WP and 0.76 in the SP. Furthermore, the gene diversity of *1-FEH w3* in the WP and SP was 0.31 and 0.36, respectively, while the PIC values for *1-FEH w3* were 0.26 and 0.30 in WP and SP, respectively.

### 2.3. Single-Marker Analysis

#### 2.3.1. Seedling Stage

Highly significant variation was found among genotypes in each population for all phenotypic traits scored [19,20]. In this study, a non-significant effect was found between the two markers and seedling traits of the WP. On the other hand, in the SP, the *TaDreb-B1* marker was not significantly associated with all traits except the SLW (*p*-value < 0.05). The *1-FEH w3* marker had a highly significant effect on all traits except DTR, with *p*-values of 0.00001, 0.00000022, and 0.0004 for SLW, DTW, and RB, respectively.

**Figure 4:**
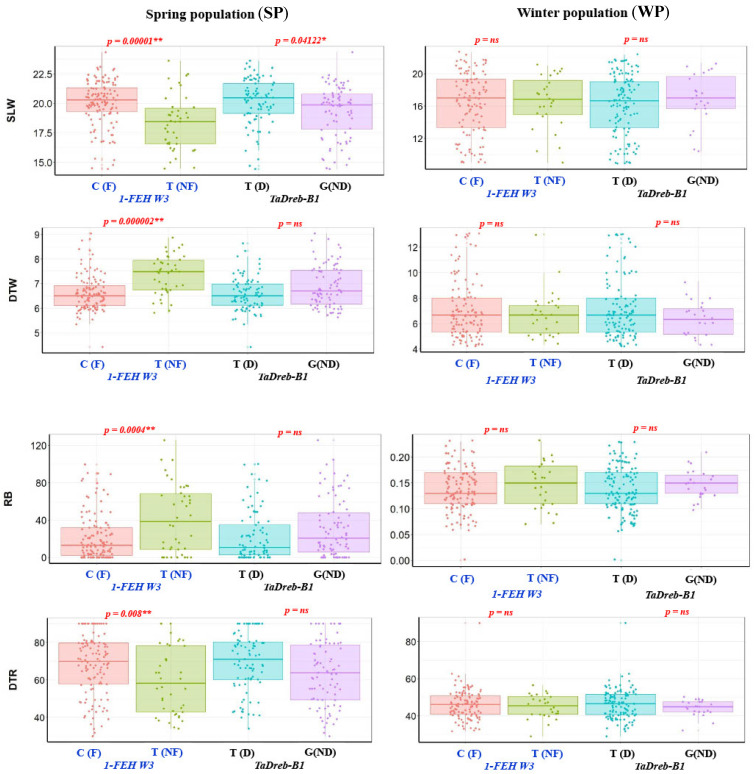
Box plots showing the difference between genotypes with the target allele and without the target allele for each marker in both populations scored at the seedling stage. F, NF, D, and ND refer to Fehw3, non-Fehw3, *TaDreb-B1*, and non-*TaDreb-B1*. (SLW) the sum of leaf witling, (DTW) days to wilting, (RB regrowth biomass), and (DTR) days to recovery. *, ** refer to significance at 0.05, and 0.01, respectively. ns refers to non-significant *p* values.

The allele effect of the two markers on the seedling traits in both populations is presented in Figure 4. In the SP, the allele C and T of the *1-FEH w3* and *Ta-Dreb-B1,* respectively, increased SLW and DTR and decreased DTW and RB. In the WP, the target allele of the *TaDreb-B1* marker had, on average, decreased SLW and RB and increased DTW and DTR. The target allele of the *1-FEH w3* in the WP, on the other hand, increased SLW, DTW, and DTR, while, it decreased RB.

#### 2.3.2. Field Experiments

Highly significant genetic variation was found among all genotypes in the winter population for all traits in the two locations in Nebraska, USA [21]. A significant difference was found between the two locations in the rainfall data (Supplementary Table S1), with averages of 3.09 cm and 1.36 cm for Lincoln and North Platte, respectively.

Highly significant variation was found for the same traits among genotypes in spring wheat (Supplementary Figures S1–S4). Soil moisture at 10 and 35 cm under drought stress in the two growing seasons are presented in Supplementary Table S2. Soil moisture (volume %) at 10 cm depth was 19.0 and 19.9% in 2018 and 2019, respectively, while it was 35.4 and 35.5% at 35 cm depth in the two growing seasons, respectively.

For the WP, the marker–trait association between the two KASP markers and the spike-related traits (SL, GNPS, SPS, and TKW) in the two distinct rainfall environments (Lincoln and North Platte) is presented in Table 1. The association between *TaDreb-B1* and spike traits in both locations was non-significant. In Lincoln, the *1-FEH w3* marker was found to be significant for all traits except TKW, with negative target allele effects on SL, GNPS, and SPS. No significant association was found between the *1-FEH w3* marker and spike traits in North Platte.

In the SP, the association between the two KASP markers and spike traits in two growth seasons (2018 and 2019) under control and drought conditions is shown in Table 2. Under control conditions, a significant association was found between SL and *TaDreb-B1* and between *1-FEH w3* and GNPS in the 2019 growing season. While under drought conditions in two seasons (2018 and 2019), *TaDreb-B1* and *1-FEH W3* were significantly associated with SL and GNPS, respectively.

## 3. Discussion

### 3.1. Diversity of KASP Markers

Improving drought tolerance in wheat remains an important task for breeders and geneticists in the face of climate change consequences, including drought stress. DNA markers can be used to accelerate breeding programs. However, they should be validated in different genetic backgrounds to be used for marker-assisted selection to improve target traits. Two KASP markers for *TaDreb-B1* and *1-FEH w3* genes were reported with their association with drought tolerance in wheat [15,16]. 

In the current study, two wheat populations were used to test the association between the two markers and drought tolerance at seedling and reproductive growth stages. The two populations facilitated reliable investigations on the effectiveness of these two markers in marker-assisted selection because they were produced differently, had different genetic backgrounds, represented two growth types of wheat (winter and spring), and each one had sufficient genotypes (>100 individuals)—recommended to test marker–trait association [22,23].

Both KASP markers were able to separate the genotypes into three clear groups (target allele, non-target allele, and heterozygous/heterogeneous). The diversity features of the two markers were extensively studied. The gene frequency of *1-FEH w3* was 0.76 and 0.81 for the spring and winter populations, respectively, which was higher than the frequency (0.28) reported by Rehman et al. [16] in a set of 153 Pakistani hexaploid wheat cultivars. For *TaDreb-B1*, the frequency of the target allele was 0.54 and 0.84 for the spring and winter populations, respectively, which was also higher than the frequency of the same allele (0.31) reported by Rehman et al. 2021. In the Pakistani hexaploid wheat cultivars, the PIC for *TaDreb-B1* and *1-FEH w3* genes was 0.33 and 0.34 [16], respectively, which were near the PIC values reported in this study for the two genes in both populations. Therefore, both markers can be classified as moderately informative (0.25 < PIC < 0.50), according to Bostein et al. [24]. So, *TaDrebB1* and *1-FEH w3* KSAP markers provided very useful information about the diversity among wheat genotypes and can also be used in diversity studies. The PIC and gene diversity values are an excellent indicator of informative markers that can be used for genetic diversity studies [25,26,27].

The association between the two target alleles for both markers and drought tolerance at seedling and adult growth stages were investigated. The same traits were recorded in both populations. Unfortunately, very few studies have investigated the effect of these two KASP markers on drought tolerance in the field. Mohamed et al. [17] tested the association between the two KASP markers and germination traits in wheat under 25 and 30% of polyethylene glycol (PEG). The association between yield traits under normal conditions and these two markers was studied by Rehman et al. [16].

### 3.2. Seedling Experiments

For the seedling experiment, SLW, DTW, RB, and DTR are very important traits that have a direct association with drought tolerance [28]. SLW and DTW are tolerance traits that indicate water deficit on wheat leaves, while RB and DTR are recovery traits indicating the ability of plants to recover and form new shoots after prolonged drought stress [29]. All these traits are highly heritable, so they are very useful for selection to drought tolerance [19,20,29,30]. In the WP, the non-significant association between the two markers and the seedling traits under drought stress indicated that there was no effect on improving drought tolerance for those genotypes that had the target allele. However, it was noted the genotypes that had allele (T) of *TaDreb-B1* had, on average, less leaf wilting symptoms, higher days to wilting, higher regrowth after drought, and lower days to recovery. The target allele (C) of *1-FEH w3* gene, on the other hand, was found to increase SLW, decrease DTW, decrease RB, and increase the DTR. Therefore, this allele can be considered, on average, to decrease drought tolerance. In the SP, the allele C of *1-FEH w3* gene was found to be highly significant and correlated with drought susceptibility for all seedling traits. The target allele of *TaDreb-B1*, on average, was non-significantly associated with seedling traits but, on average, the genotypes possessing this allele had high SLW, high DTW, low DTR, and low RB. Germination is a growth stage before the seedling stage. No significant association was identified between the two KASP markers and germination traits (germination %, germination rate, shoot length, root length, number of roots, fresh and dry weight, and water content) under severe drought stress induced by PEG [17]. Additionally, no clear allele effect, on average, regarding drought tolerance, was found for all traits under both PEG treatments.

### 3.3. Field Experiments

For the field experiment, both populations were evaluated under normal irrigation (SP) or high-rainfall (WP) and drought or low-rainfall conditions in two different countries (Egypt and USA). The significant differences in the WP between the two locations in the rainfall indicated that the genotypes at North Platte were exposed to greater drought stress compared to those grown in Lincoln. For the spring population, the soil moisture (volume %) was approximately 19% and 35% at 10 and 35 cm depth. In wheat, the optimum volumetric soil moisture content remaining at field capacity was about 45 to 55% (three feet below the soil surface for clay soils and 15 to 20% at the wilting point [31], which is defined as the soil water content when plants growing in that soil wilt and fail to recover their turgor upon rewetting, indicating successful drought stress occurred in spring genotypes).

Under normal (SP) and high-rainfall (WP) conditions, the target allele (C) for the *1-FEH w3* was found to be significantly associated with low GNPS (C19), while in the spring population, it was associated with low SPS, low GNPS and short SL (Lincoln) in the winter population. Additionally, it had a negative effect, on average, on TKW in both populations. These results agreed with previous results reported by Rehman et al. [16], who found that the favorable allele (C) of *1-FEH w3* was significantly associated with decreased grain number per spike (GPS). They also found a non-significant association between the *1-FEH w3* marker and TKW; however, in both populations, in the genotypes that had the C allele, TKW decreased compared to the genotypes that carried the other allele (T). For the *TaDreb-B1* marker, the target allele (T) was significantly associated with decreased spike length. In the study of Rehman et al. [16], the target allele of the *TaDreb-B1* was significantly associated with increased grain number per spike. In the current study, all genotypes in both populations carrying the target allele (T) of the *TaDreb-B1* had, on average, higher GNPS and SPS than the group with the nontarget allele (C). The target allele (T) decreased TKW in both populations. Although Rheman et al. [16] reported a non-significant association between the *TaDreb-B1* and TKW, on average, the genotypes carrying the target allele had higher TKW than the other group.

Under low-rainfall and drought stress conditions, the allele C of the *1-FEH w3* gene was significantly associated with increased GNPS in D18 and decreased GNPS in D19 in the spring population, indicating an environmentally sensitive effect on GNPS across the two years under drought stress. The same markers did not show a significant association in the winter population under low-rainfall environments. The *TaDreb-B1* markers had a significant association with decreased SL in D18 and D19 in the spring population. On average, the target allele of the *TaDreb-B1 (T)* increased SPS and GNPS (except D19) in both populations. Unfortunately, no previous study investigated the marker–trait association between markers and yield traits under drought stress conditions.

**Figure 5:**
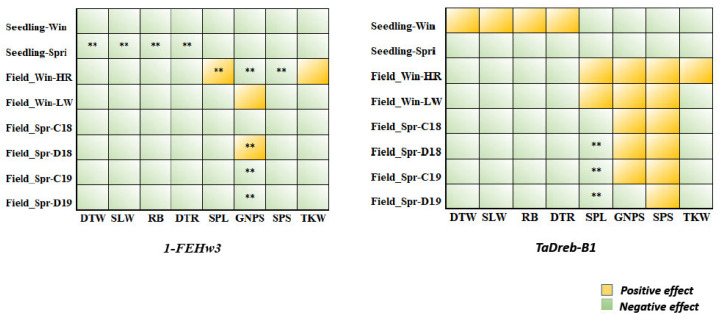
Target allele effect of each gene in all traits scored in spring and winter populations. ** Refers to significant association at *p* > 0.01. (SLW) the sum of leaf witling, (DTW) days to wilting, (RB regrowth biomass), and (DTR) days to recovery, (SL) Spike length, (SPS) number of spikelets per spike, (GNPS) grain number per spike, and (TKW) thousand-kernel weight.

To summarize the effects of two markers in both populations in the current study, the allele effect (on average) of the target alleles of each marker for each trait is illustrated in Figure 5. In total, it seemed that *TaDreb-B1* had more positive effects in most of the traits at the seedling stage and adult growth stages than the *1-FEH w3* marker (four traits). Notably, on average, all genotypes carrying the target allele (T) of *TaDreb-B1* had higher GNPS (except D19) and SPS than non-allele genotypes under both conditions in both populations. The findings of Rehman et al. [16] (under normal conditions) support this result. Therefore, the *TaDreb-B1* marker might be more effective in MAS than the *1-FEH w3* marker.

The SNP alleles of *1-FEH w3* were reported by Zhang et al. [32]. The flanking sequence including the target allele (C) was blasted in *EnsemblePlant* (https://plants.ensembl.org/Triticum_aestivum/Info/Index (accessed on 15 May 2023). The blast results revealed that the sequence was located within the TraesCS6B02G080700 gene model, which is known as *1-FEH w3* (https://knetminer.org/Triticum_aestivum/ (accessed on 15 May 2023). Zhang et al. [32] tested the expression of *1-FEH w3* in two contrasting wheat genotypes for drought tolerance. They found that *1-FEH w3* had a higher expression in the drought tolerant genotype than in the drought susceptible genotype. The tolerant genotype had the allele C, while the susceptible genotype had the T allele. This marker segregated very well in the doubled haploid population (DH) derived from the crossing of two genotypes. In the DH population, the *1-FEH w3* allele (C) was found to be significantly associated with high thousand-grain weight under drought and well-watered conditions. Additionally, the target allele (C) of *1-FEH w3* had a non-significant association with kernel number per spike. The DH population only exhibited the variation observed between the two parents. But the diverse populations provide more genetic variation and diversity, making them ideal for identifying alleles and their real effects using marker–trait association analysis [33,34]. In a study by Yáñez et al. [35], the expression of *1-FEH w3* gene was studied in eight contrasting wheat genotypes that were selected based on the drought tolerance index. They found that the *1-FEH w3* gene had a higher expression in the susceptible genotype Fontagro 69 than the tolerant genotype LE2384 at 0, 14, and 21 days after anthesis under water stress. Moreover, the susceptible genotype had a higher thousand kernel weight under normal and drought stress than the tolerant genotype and vice versa for the number of kernels per spike, number of spikelets per spike, and grain yield per plant. This result further supports that *1-FEH w3* as a KASP marker had a non-stable effect on drought tolerance in wheat. As drought tolerance is a complex trait controlled by many genes, it seems that the target allele for the *Fewh3* gene may have only small effects on drought tolerance in wheat. It could be that the other genotypes that did not have the target allele include other drought-tolerant genes. In wheat, the negative and non-significant effect of the C allele of the *1*-*FEWHw3* gene was found at the germination stage [17] and also at the adult growth stage in a large number of highly diverse wheat genotypes [16]. 

KASP markers are very important for marker-assisted selection to rapidly and genetically target traits [36]. Therefore, KASP markers should be validated in a large number of genotypes with high genetic diversity before use in MAS.

## 4. Material and Methods

### 4.1. Plant Material

The plant material used in this study consisted of two wheat populations (winter and spring). The winter population consisted of 157 randomly selected genotypes from the 270 F _3:6_ lines (Nebraska Duplicate Nursery 2017). Details about the production and pedigree of the population were previously published in Eltaher et al. [21]. All genotypes in the winter population (WP) were highly diverse, representing the preliminary yield trial of a standard breeding program [27]. The spring population (SP) consisted of 198 highly diverse wheat genotypes, representing 22 different countries [19]. The list of genotypes in both populations is presented in Supplementary Table S3. The genotypes from SP were collected from the U.S. National Plant Germplasm System database (https://www.ars-grin.gov/ (accessed on 15 May 2023), while the genotypes in WP were from Nebraska Winter Wheat Breeding program (https://agronomy.unl.edu/small-grains (accessed on 15 May 2023).

### 4.2. Seedlings Experiments

Both populations were previously evaluated under seedling drought stress using the same protocol with very slight modifications in the duration of drought stress. Drought tolerance was evaluated in the spring population by Ahmed et al. [19], while the winter population was evaluated by Eltaher et al. [21].

In both populations, all genotypes were exposed to natural drought stress by water withholding after all plants reached two expanded leaves in the winter population and one expanded leaf in the spring. When 70% of genotypes had full wilting, the drought treatment was ended. Then, all plants were cut and re-irrigated to test their recovery after prolonged drought stress. Four traits were selected from Ahmed et al. [19]. These traits were the sum of leaf witling (SLW), days to wilting (DTW), regrowth biomass (RB), and days to recovery (DTR). Low values of SLW and DTR and high values of RB and DTW indicated tolerance to drought. These traits have a direct relation to seedling drought stress.

The genotypes in spring and winter population were evaluated in seven and three replications, respectively.

### 4.3. Field Experiments

Important spike traits in the winter population were scored under a higher-rainfall environment (HR) located in Lincoln, NE, and a lower-rainfall environment (LW) located in North Platte, NE, USA by Eltaher et al. [21] in two replications each. The climate data, including temperature and precipitation for the two locations, are presented in Supplementary Table S1.

In this study, the spring population was evaluated under normal and drought stress conditions for two growing seasons (2018 and 2019) at the Experimental Field Station, Assiut University, Egypt, where soil and clay were used in two replications in each of the conditions. In each growing season, the genotypes in the control conditions were irrigated eight times during the growing season, while the irrigation was stopped after two times irrigation at the booting stage in the drought conditions.

Spike length (SL), number of spikelets per spike (SPS), grain number per spike (GNPS), and thousand-kernel weight scored in the spring population, and the data of the same traits were taken from Eltaher et al. [21] for the winter population. Soil water moisture was estimated under drought stress at 10 and 35 cm depth according to method described by Kumar [37].

### 4.4. DNA Extraction and Kompetitive Allele-Specific PCR (KASP) Genotyping

All genotypes in both populations were sown, and when all plants reached the one leaf stage, the leaves of each genotype were harvested for DNA extraction. All genotypes in the spring and winter populations are highly homozygous. DNA extraction was performed using the BioSprint 96 automatic DNA extractor. For KASP genotyping, the DNA concentrations of all samples were diluted at 50 ng/µL according to LGC Genomics manual instructions. All samples were arrayed in a 384-well plate for KASP PCR. In each well, a 10 µL reaction (5 µL of DNA and 5 µL of the KASP reaction mix, including 0.14 µL of the primer assay mix from LGC-Genomics, Middlesex, UK) was used.

Two KASP markers for *TaDreb-B1* and *1-1-FEH w3* genes were ordered from the Integrated DNA Technologies (IDT) laboratory (Redwood City, CA, USA) (Supplementary Table S4). Thermal cycling conditions lasted 15 min at 94 °C. This was followed by 10 cycles of touchdown PCR as follows: 94 °C for 20 s and 65–57 °C for 60 s (dropping to 0.8 °C per cycle). This was followed by 26 cycles of regular PCR as follows: 94 °C for 20 s and 55 °C for 60 s. The 384-well plate was read via FLUOstar Omega fluorescence. To determine the absence or presence of the *TaDreb-B1* and *1-FEH w3* genes, the target allele (presence of gene) was labelled with FAM (blue), while the non-target allele A (absence of gene) was labelled with HEX (red). The analysis of KASP-PCR products was performed using Klustercaller v2.22.0.5 software, and SNPs of each gene were labelled using SNPviewer software (LGC, Biosearch Technologies, Beverly, MA, USA). The analysis of KASP markers in the two populations was done in 2022. 

### 4.5. Diversity and Single-Marker Analysis

In each population, the polymorphic information content, gene diversity, and gene frequency were estimated for each KASP marker using Power marker software [38].

To test marker–trait association, the SNPs of each gene were analyzed with all traits scored in this study in both populations by single-marker analysis (SMA). The SMA was performed using R software according to the following model [39]:

*Y* = *µ* + *f* (*marker*) + *error*

where *Y* is the trait value, *µ* is the nursery mean, and *f* (marker) is a function of the molecular marker [40].

The significant marker–trait association was determined to be at a significant level of *p* < 0.05. The phenotypic variation explained by marker and gene effect was calculated from the statistical model [39]. Box plots showing the difference between genotypes having the target allele and without the target allele were created using the R package ‘ggplot2’ [41].

## 5. Conclusions

In conclusion, two highly diverse populations representing winter and spring types were used to investigate the effectiveness of two KASP markers, *1-FEH w3* and *TaDreb-B1*, on improving drought tolerance in wheat for MAS. The results of this study suggest that the use of *1-FEH w3* as a KASP marker for MAS to improve drought tolerance in wheat is extremely questionable. *TaDreb-B1* provided better consistency in improving drought tolerance than *1-FEH w3*. However, the promising marker allele *TaDreb-B1* should be validated further in different genetic backgrounds.

## Figures and Tables

**Table 1:** Marker–trait association analysis between the two KASP markers and spike traits under Lincoln and North Platte (low rainfall environment) in the winter population.

Location	Trait	Gene/Target Allele	*p*-Value	Effect of Target Allele
Lincoln	SL	*1-FEH w3*/C	0.01727 **	−0.38931
		*TaDreb-B1*/T	0.30918	0.17946
	GNPS	*1-FEH w3*/C	0.00517 **	−3.2963
		*TaDreb-B1*/T	0.66216	0.56407
	SPS	*1-FEH w3*/C	0.03281 **	−0.53918
		*TaDreb-B1*/T	0.95199	0.01643
	TKW	*1-FEH w3*/C	0.78477	0.44653
		*TaDreb-B1*/T	0.44112	−0.17361
	SL	*1-FEH w3*/C	0.50759	0.08685
		*TaDreb-B1*/T	0.53501	0.08919
	GNPS	*1-FEH w3*/C	0.60044	0.34391
North Platte		*TaDreb-B1*/T	0.63706	0.33435
	SPS	*1-FEH w3*/C	0.79417	−0.0495
		*TaDreb-B1*/T	0.89495	0.0268
	TKW	*1-FEH w3*/C	0.48396	−0.3992
		*TaDreb-B1*/T	0.16237	−0.8338

(SL) Spike length, (SPS) number of spikelets per spike, (GNPS) grain number per spike, and (TKW) thousand-kernel weight. ** refers to significance at *p ≤* 0.01.

**Table 2:** Marker–trait association analysis between the two KASP markers and spike traits under control and drought conditions in two growing seasons (2018 and 2019) in the spring population.

Environment	Trait	Gene/Target Allele	*p*-Value	Effect of Target Allele
Control 2018				
	SL	*1-FEH w3*/C	0.647	−0.307
		*TaDreb-B1*/T	0.105	−0.882
	GNPS	*1-FEH w3*/C	0.143	−5.190
		*TaDreb-B1*/T	0.169	4.115
	SPS	*1-FEH w3*/C	0.941	−0.033
		*TaDreb-B1*/T	0.788	0.103
	TKW	*1-FEH w3*/C	0.852	−0.297
		*TaDreb-B1*/T	0.461	−1.002
Drought 2018				
	SL	*1-FEH w3*/C	0.371	−0.381
		*TaDreb-B1*/T	0.000096 **	−1.393
	GNPS	*1-FEH w3*/C	0.021180 *	3.464
		*TaDreb-B1*/T	0.191	5.059
	SPS	*1-FEH w3*/C	0.831	−0.078
		*TaDreb-B1*/T	0.613	0.152
	TKW	*1-FEH w3*/C	0.966	−0.073
		*TaDreb-B1*/T	0.330	−1.382
Control 2019				
	SL	*1-FEH w3*/C	0.500	−0.358
		*TaDreb-B1*/T	0.00037 **	−1.540
	GNPS	*1-FEH w3*/C	0.02732 *	−6.738
		*TaDreb-B1*/T	0.836	0.519
	SPS	*1-FEH w3*/C	0.420	−0.374
		*TaDreb-B1*/T	0.523	0.251
	TKW	*1-FEH w3*/C	0.503	−0.455
		*TaDreb-B1*/ T	0.082	−0.942
Drought 2019				
	SL	*1-FEH w3*/C	0.651	−0.198
		*TaDreb-B1*/T	0.00051 **	−1.246
	GNPS	*1-FEH w3*/C	0.00811 **	−8.161
		*TaDreb-B1*/T	0.524	-d1.588
	SPS	*1-FEH w3*/C	0.356	−0.377
		*TaDreb-B1*/T	0.201	0.444
	TKW	*1-FEH w3*/C	0.802	−0.358
		*TaDreb-B1*/T	0.546	−0.718

(SL) Spike length, (SPS) number of spikelets per spike, (GNPS) grain number per spike, and (TKW) thousand-kernel weight, *, ** significant at *p ≤* 0.05 and *p ≤* 0.01, respectively.

## Data Availability

All data generated or analyzed during this study are included in this published article and its supplementary information files.

## Supplementary Materials

The following supporting information can be downloaded at: https://www.mdpi.com/article/10.3390/ijms24108986/s1.
