# Supplementary material for: Effectiveness of TaDreb-B1 and 1-FEH w3 KASP Markers in Spring and Winter Wheat Populations for Marker-Assisted Selection to Improve Drought Tolerance
Source: Int J Mol Sci. 2023 May 19;24(10):8986. doi: 10.3390/ijms24108986 (PMC10218890; doi:10.3390/ijms24108986)
Supplement: Supplementary file 1 [file ijms-24-08986-s001.zip › Supp Figures.pptx]

## Slide 1
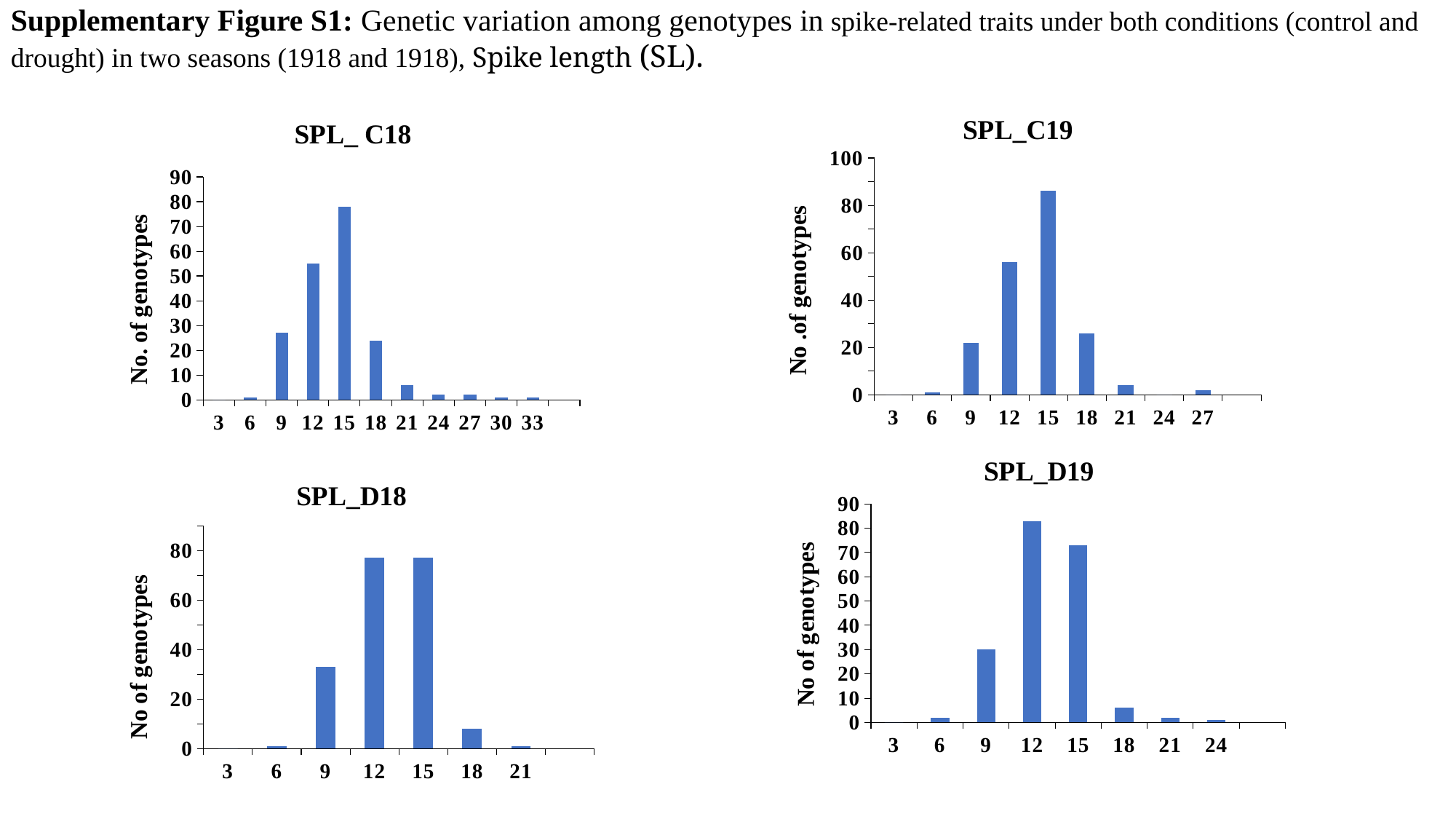

Supplementary Figure S1: Genetic variation among genotypes in spike-related traits under both conditions (control and drought) in two seasons (1918 and 1918), Spike length (SL).
### Chart: SPL_C19
| Category | |
|---|---|
| 3 | 0.0 |
| 6 | 1.0 |
| 9 | 22.0 |
| 12 | 56.0 |
| 15 | 86.0 |
| 18 | 26.0 |
| 21 | 4.0 |
| 24 | 0.0 |
| 27 | 2.0 |
| | None |
### Chart: SPL_ C18
| Category | |
|---|---|
| 3 | 0.0 |
| 6 | 1.0 |
| 9 | 27.0 |
| 12 | 55.0 |
| 15 | 78.0 |
| 18 | 24.0 |
| 21 | 6.0 |
| 24 | 2.0 |
| 27 | 2.0 |
| 30 | 1.0 |
| 33 | 1.0 |
| | None |
### Chart: SPL_D19
| Category | |
|---|---|
| 3 | 0.0 |
| 6 | 2.0 |
| 9 | 30.0 |
| 12 | 83.0 |
| 15 | 73.0 |
| 18 | 6.0 |
| 21 | 2.0 |
| 24 | 1.0 |
| | None |
### Chart: SPL_D18
| Category | |
|---|---|
| 3 | 0.0 |
| 6 | 1.0 |
| 9 | 33.0 |
| 12 | 77.0 |
| 15 | 77.0 |
| 18 | 8.0 |
| 21 | 1.0 |
| | None |

## Slide 2
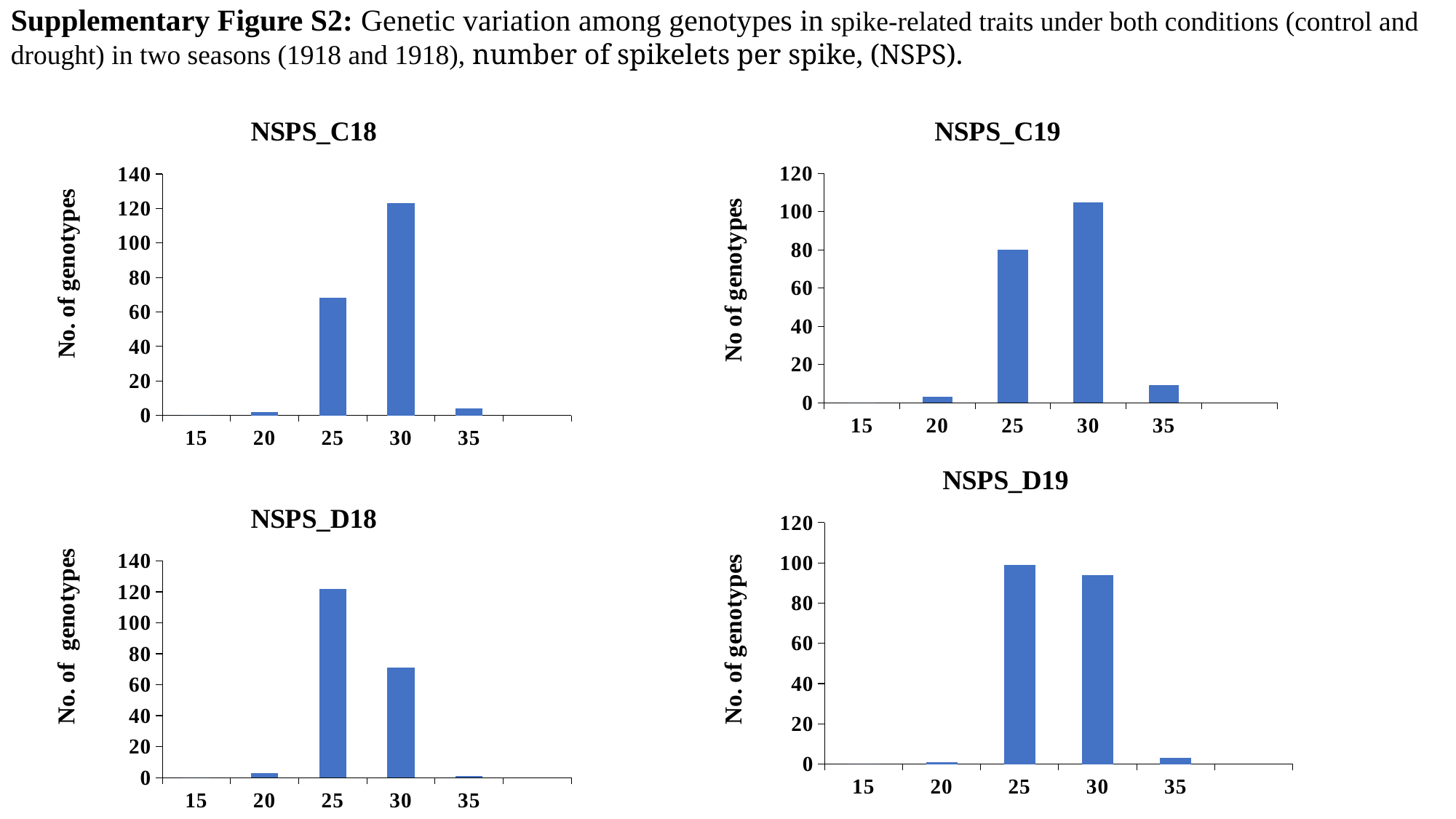

Supplementary Figure S2: Genetic variation among genotypes in spike-related traits under both conditions (control and drought) in two seasons (1918 and 1918), number of spikelets per spike, (NSPS).
### Chart: NSPS_C18
| Category | |
|---|---|
| 15 | 0.0 |
| 20 | 2.0 |
| 25 | 68.0 |
| 30 | 123.0 |
| 35 | 4.0 |
| | None |
### Chart: NSPS_C19
| Category | |
|---|---|
| 15 | 0.0 |
| 20 | 3.0 |
| 25 | 80.0 |
| 30 | 105.0 |
| 35 | 9.0 |
| | None |
### Chart: NSPS_D19
| Category | |
|---|---|
| 15 | 0.0 |
| 20 | 1.0 |
| 25 | 99.0 |
| 30 | 94.0 |
| 35 | 3.0 |
| | None |
### Chart: NSPS_D18
| Category | |
|---|---|
| 15 | 0.0 |
| 20 | 3.0 |
| 25 | 122.0 |
| 30 | 71.0 |
| 35 | 1.0 |
| | None |

## Slide 3
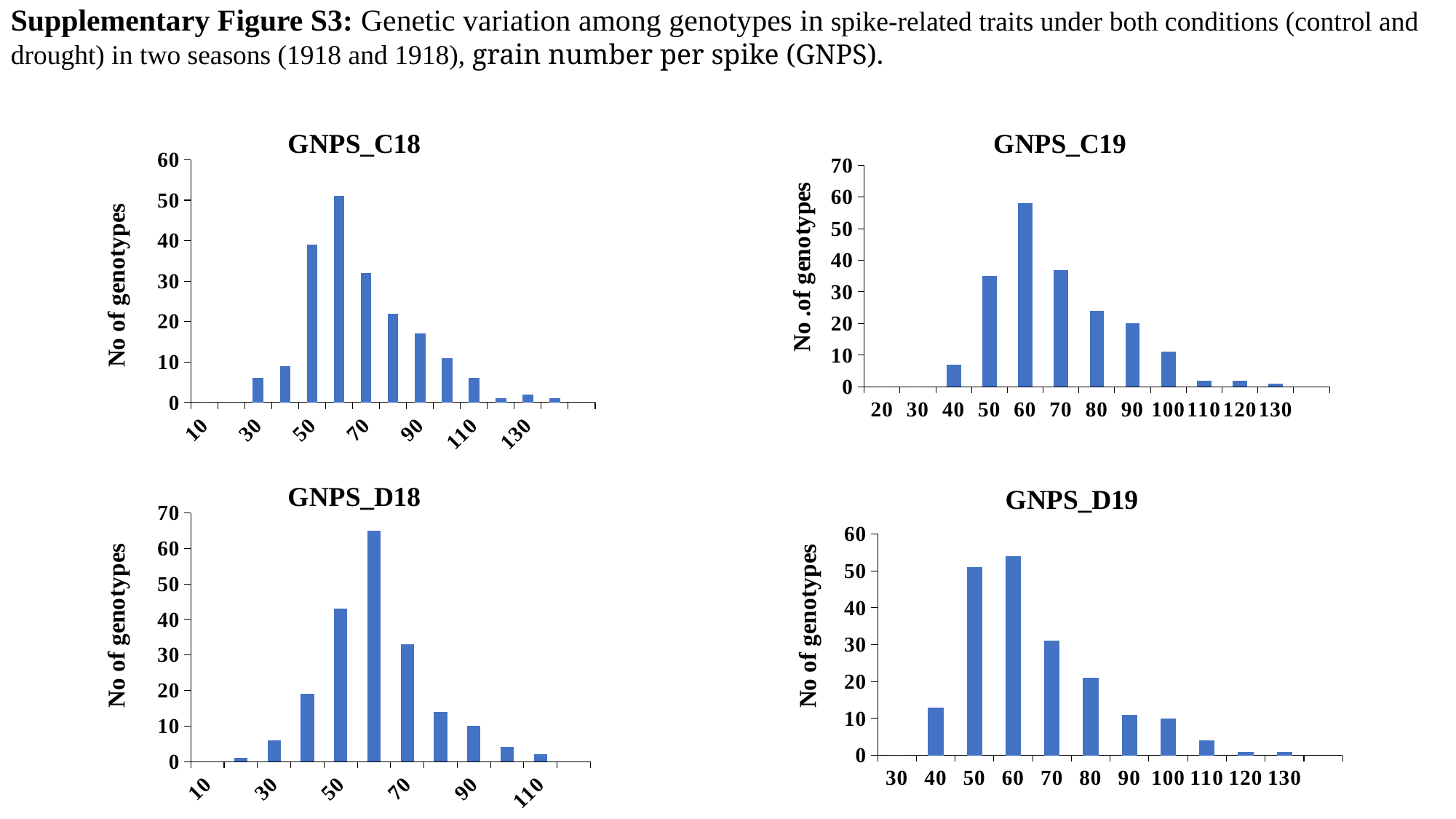

Supplementary Figure S3: Genetic variation among genotypes in spike-related traits under both conditions (control and drought) in two seasons (1918 and 1918), grain number per spike (GNPS).
### Chart: GNPS_C18
| Category | |
|---|---|
| 10 | 0.0 |
| 20 | 0.0 |
| 30 | 6.0 |
| 40 | 9.0 |
| 50 | 39.0 |
| 60 | 51.0 |
| 70 | 32.0 |
| 80 | 22.0 |
| 90 | 17.0 |
| 100 | 11.0 |
| 110 | 6.0 |
| 120 | 1.0 |
| 130 | 2.0 |
| 140 | 1.0 |
| | None |
### Chart: GNPS_C19
| Category | |
|---|---|
| 20 | 0.0 |
| 30 | 0.0 |
| 40 | 7.0 |
| 50 | 35.0 |
| 60 | 58.0 |
| 70 | 37.0 |
| 80 | 24.0 |
| 90 | 20.0 |
| 100 | 11.0 |
| 110 | 2.0 |
| 120 | 2.0 |
| 130 | 1.0 |
| | None |
### Chart: GNPS_D18
| Category | |
|---|---|
| 10 | 0.0 |
| 20 | 1.0 |
| 30 | 6.0 |
| 40 | 19.0 |
| 50 | 43.0 |
| 60 | 65.0 |
| 70 | 33.0 |
| 80 | 14.0 |
| 90 | 10.0 |
| 100 | 4.0 |
| 110 | 2.0 |
| | None |
### Chart: GNPS_D19
| Category | |
|---|---|
| 30 | 0.0 |
| 40 | 13.0 |
| 50 | 51.0 |
| 60 | 54.0 |
| 70 | 31.0 |
| 80 | 21.0 |
| 90 | 11.0 |
| 100 | 10.0 |
| 110 | 4.0 |
| 120 | 1.0 |
| 130 | 1.0 |
| | None |

## Slide 4
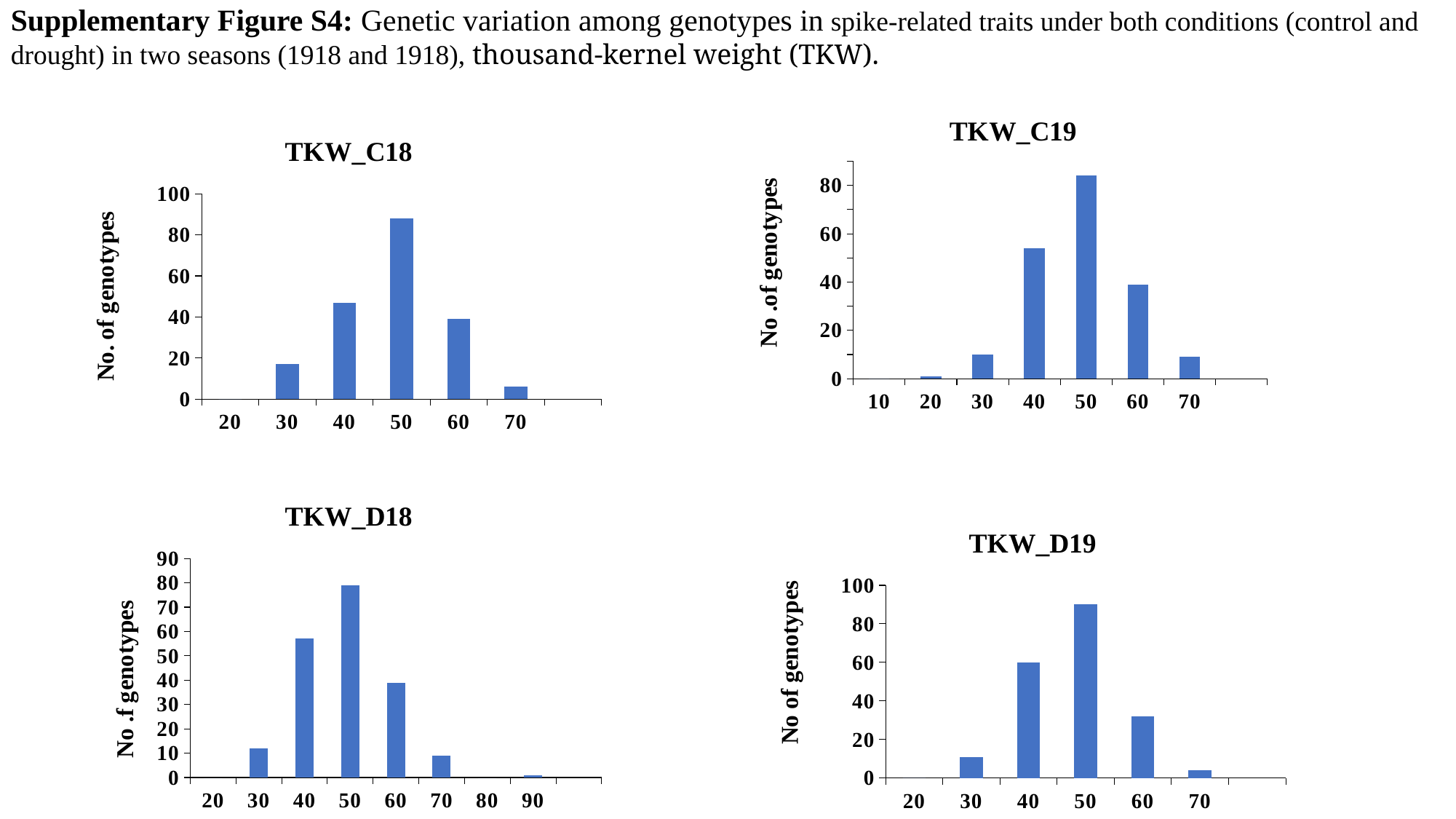

Supplementary Figure S4: Genetic variation among genotypes in spike-related traits under both conditions (control and drought) in two seasons (1918 and 1918), thousand-kernel weight (TKW).
### Chart: TKW_C19
| Category | |
|---|---|
| 10 | 0.0 |
| 20 | 1.0 |
| 30 | 10.0 |
| 40 | 54.0 |
| 50 | 84.0 |
| 60 | 39.0 |
| 70 | 9.0 |
| | None |
### Chart: TKW_C18
| Category | |
|---|---|
| 20 | 0.0 |
| 30 | 17.0 |
| 40 | 47.0 |
| 50 | 88.0 |
| 60 | 39.0 |
| 70 | 6.0 |
| | None |
### Chart: TKW_D18
| Category | |
|---|---|
| 20 | 0.0 |
| 30 | 12.0 |
| 40 | 57.0 |
| 50 | 79.0 |
| 60 | 39.0 |
| 70 | 9.0 |
| 80 | 0.0 |
| 90 | 1.0 |
| | None |
### Chart: TKW_D19
| Category | |
|---|---|
| 20 | 0.0 |
| 30 | 11.0 |
| 40 | 60.0 |
| 50 | 90.0 |
| 60 | 32.0 |
| 70 | 4.0 |
| | None |
